# Cholinesterase Inhibitors and Hospitalization for Bradycardia: A Population-Based Study

**DOI:** 10.1371/journal.pmed.1000157

**Published:** 2009-09-29

**Authors:** Laura Y. Park-Wyllie, Muhammad M. Mamdani, Ping Li, Sudeep S. Gill, Andreas Laupacis, David N. Juurlink

**Affiliations:** 1 Department of Family and Community Medicine, St. Michael's Hospital, Toronto, Ontario, Canada; 2 Keenan Research Centre in the Li Ka Shing Knowledge Institute of St. Michael's Hospital, Toronto, Ontario, Canada; 3 Institute for Clinical Evaluative Sciences, Toronto, Ontario, Canada; 4 Leslie Dan Faculty of Pharmacy, University of Toronto, Toronto, Ontario, Canada; 5 Department of Health Policy, Management and Evaluation, University of Toronto, Toronto, Ontario, Canada; 6 Department of Medicine at Queens University, Kingston, Ontario, Canada; 7 Department of Medicine, University of Toronto, Toronto, Ontario, Canada; 8 Department of Medicine, Sunnybrook Health Sciences Centre, Toronto, Ontario, Canada; University of Cambridge, United Kingdom

## Abstract

**Background:**

Cholinesterase inhibitors are commonly used to treat dementia. These drugs enhance the effects of acetylcholine, and reports suggest they may precipitate bradycardia in some patients. We aimed to examine the association between use of cholinesterase inhibitors and hospitalization for bradycardia.

**Methods and Findings:**

We examined the health care records of more than 1.4 million older adults using a case-time-control design, allowing each individual to serve as his or her own control. Case patients were residents of Ontario, Canada, aged 67 y or older hospitalized for bradycardia between January 1, 2003 and March 31, 2008. Control patients (3∶1) were not hospitalized for bradycardia, and were matched to the corresponding case on age, sex, and a disease risk index. All patients had received cholinesterase inhibitor therapy in the 9 mo preceding the index hospitalization. We identified 1,009 community-dwelling older persons hospitalized for bradycardia within 9 mo of using a cholinesterase inhibitor. Of these, 161 cases informed the matched analysis of discordant pairs. Of these, 17 (11%) required a pacemaker during hospitalization, and six (4%) died prior to discharge. After adjusting for temporal changes in drug utilization, hospitalization for bradycardia was associated with recent initiation of a cholinesterase inhibitor (adjusted odds ratio [OR] 2.13, 95% confidence interval [CI] 1.29–3.51). The risk was similar among individuals with pre-existing cardiac disease (adjusted OR 2.25, 95% CI 1.18–4.28) and those receiving negative chronotropic drugs (adjusted OR 2.34, 95% CI 1.16–4.71). We found no such association when we replicated the analysis using proton pump inhibitors as a neutral exposure. Despite hospitalization for bradycardia, more than half of the patients (78 of 138 cases [57%]) who survived to discharge subsequently resumed cholinesterase inhibitor therapy.

**Conclusions:**

Among older patients, initiation of cholinesterase inhibitor therapy was associated with a more than doubling of the risk of hospitalization for bradycardia. Resumption of therapy following discharge was common, suggesting that the cardiovascular toxicity of cholinesterase inhibitors is underappreciated by clinicians.

*Please see later in the article for the Editors' Summary*

## Abstract 2

Laura Park-Wyllie and colleagues examined the health records of more than 1.4 million older adults and show that initiation of cholinesterase inhibitor therapy is associated with a more than doubling of the risk of hospitalization for bradycardia.

## Editors' Summary

**Background:**

Alzheimer disease and other forms of dementia principally affect people aged over 65. These conditions result in confusion, long term memory loss, irritability, and mood swings. As the population of developed countries ages, the prevalence of dementia is expected to increase significantly. It is forecast that the proportion of people with dementia in the US will quadruple by 2045.

A common treatment for Alzheimer disease is a class of drug called an acetylcholinesterase inhibitor or cholinesterase inhibitor. These include donepezil (brand name Aricept), rivastigmine (marketed as Exelon and Exelon Patch), and galantamine (branded Razadyne).

The benefit of taking cholinesterase inhibitors is generally small and they cannot reverse the effects of dementia. In about 50% of patients they delay the worsening of symptoms for between six months and a year, although a small number of patients may benefit more. They can have unpleasant side effects, which may include diarrhoea and muscle cramps.

**Why Was This Study Done?:**

Existing evidence is inconclusive on whether cholinesterase inhibitors increase the risk of bradycardia, an abnormally slow resting heart rate of below 60 beats a minute, which can cause fatigue, dizziness, fainting, palpitations, shortness of breath, or death. In this paper, the authors use routinely collected health care data to investigate whether an older person taking a cholinesterase inhibitor is at increased risk of bradycardia.

**What Did the Researchers Do and Find?:**

They began by supposing that cholinesterase inhibitors might induce bradycardia soon after a patient first began to take them. To investigate this, they obtained health care data on 1.4 million patients aged 67 or over in Ontario, Canada. They identified 161 patients who had visited a hospital for bradycardia and who had previously taken a cholinesterase inhibitor only within specific periods of time. They found that 139 had taken a cholinesterase inhibitor within the previous three months compared with 22 who had stopped taking it at least six months before.

They compared these cases with up to three “control” patients who matched each of the initial “case” group of 161 patients by age, sex, and risk of bradycardia on the basis of their general health. None of the 466 controls had visited a hospital for bradycardia by the “index date,” that is, the date of hospitalization of the case patient they matched. The researchers found 349 of the control patients had begun to take a cholinesterase inhibitor in the three months prior to the index date, compared with 117 who had stopped taking it at least six months before. A statistical analysis of these data showed that recent initiation of cholinesterase inhibitors was associated with approximately a doubling of the risk of hospitalization for bradycardia.

The authors repeated their procedure to see whether another class of drug, proton pump inhibitors, had a similar effect. As they had expected, it did not. They repeated the analysis for patients taking into account other drugs that slow the heart rate and found that their increased risk of bradycardia when taking a cholinesterase inhibitor persisted. The increase in risk was also similar in patients with pre-existing heart problems.

The researchers' data also showed that, excluding patients who while in the hospital had a pacemaker fitted to control their heart rate, over half of the patients released from hospital started taking a cholinesterase inhibitor again. Of these, a few returned to hospital with bradycardia within 100 days.

**What Do These Findings Mean?:**

Recent guidelines suggest that doctors should not prescribe cholinesterase inhibitors for dementia patients as a matter of course, but weigh the potential risks and benefits. This paper provides evidence of an additional risk, of which at least some doctors are unaware. It was not possible to compare risk for different cholinesterase inhibitors because most patients took donepezil.

A population-based study like this cannot prove that cholinesterase inhibitors cause bradycardia. The authors used routinely collected data and so did not have information on all relevant risk factors, and thus there remains a possibility of bias due to unmeasured factors. In addition the authors had to make assumptions, for instance that patients took the drugs prescribed for them. They also considered only diagnoses of bradycardia made by a hospital doctor and not those made elsewhere, which means the incidence of bradycardia may have been underestimated. A strength of the study is the use of a case-time-control design, which has the advantage of reducing bias due to the different health conditions and lifestyle of individual patients, and also bias due to factors changing over time.

**Additional Information:**

Please access these Web sites via the online version of this summary at http://dx.doi.org/10.1371/journal.pmed.1000157.

- Wikipedia contains information on Alzheimer disease (note that Wikipedia is a free online encyclopedia that anyone can edit; available in several languages)
- Information on bradycardia and its causes can be found in Wikipedia (note that Wikipedia is a free online encyclopedia that anyone can edit; available in several languages)
- The UKs National Health Service provides information on dementia, including symptoms, causes, diagnosis, treatment, and prevention
- MedlinePlus provides US-based health information (in English and Spanish)
- The US National Institute on Aging provides information on health, relevant to older people, including Alzheimer Disease and dementia (in English and Spanish)
- The US Alzheimers Association contains useful information on the disease, including on medication
- The Public Health Agency of Canada website provides information on senior health (in English and French)
- The UK-based Alzheimers Society provides advice on caring for people with dementia

## Introduction

Cholinesterase inhibitors such as donepezil, rivastigmine, and galantamine are widely prescribed to improve cognitive function in patients with Alzheimer disease—a condition expected to quadruple in prevalence over the next 50 y [1]. By inhibiting the synaptic metabolism of acetylcholine, these drugs enhance cortical cholinergic neurotransmission [2]. Although cholinesterase inhibitors are generally well tolerated, they may provoke adverse effects in some patients because their cholinergic effects are not confined to the central nervous system [2]. Symptoms of cholinergic excess are often nonspecific and include gastrointestinal upset, diarrhea, hypersalivation, and muscle cramps. In severe instances, these drugs can increase vagal tone and thereby precipitate bradycardia.

Anecdotal reports, small observational studies, and post hoc analyses of clinical trials have produced conflicting results, with some suggesting an increased risk of bradycardia during cholinesterase inhibitor therapy and others finding no such association [3]–[12]. At the time of our study, no large-scale studies had examined, to our knowledge, whether cholinesterase inhibitor use among older patients predisposes to bradycardia.

Frail older adults represent a growing population of cholinesterase inhibitor users. These patients are more prone to the adverse effects of drugs and discontinue cholinesterase inhibitors more often than patients in clinical trials, who are typically healthier than those in clinical practice [10]. We sought to characterize the association between cholinesterase inhibitor therapy and hospitalization for bradycardia in a population of more than 1.4 million older adults.

## Methods

### Setting and Data Sources

We linked multiple population-based health care databases in an anonymous fashion using unique encrypted health card numbers. This linkage process has been standardized by our research institution (http://www.ices.on.ca), and these methods have been used extensively to study population-based health outcomes, including adverse drug events [13]–[20]. The Ontario Drug Benefit database was used to identify prescription records, and contains comprehensive, high-quality information regarding prescription medications dispensed to Ontario residents aged 65 y and older [21]. The Canadian Institute for Health Information (CIHI) Discharge Abstract Database was used to identify hospital admissions, and contains detailed diagnostic and procedural information for all hospital admissions in Ontario. The National Ambulatory Care Reporting System was used to identify visits to emergency departments. Basic demographic information was obtained from the Ontario Registered Persons Database. Finally, we used the Ontario Health Insurance Plan database to identify claims for inpatient and outpatient physician services. All Ontario seniors receive universal access to hospital care, physicians' services, and prescription drug coverage. The study was approved by the Research Ethics Board of Sunnybrook Health Sciences Centre.

### Study Design

We used the case-time-control design to examine the association between cholinesterase inhibitor use and hospitalization for bradycardia among Ontario residents aged 67 y and older. This design is an extension of the case-crossover design first described by Maclure [22],[23], which compares within-patient exposure to a potential risk factor in the period immediately preceding a putative adverse event (the risk interval) to exposure during a different time (the reference interval). Because cases serve as their own controls, fixed patient characteristics are controlled for implicitly under this design [24],[25]. However, the case-crossover design can be vulnerable to spurious associations between a drug and an outcome owing to temporal trends in drug utilization. The case-time-control design corrects for this limitation by incorporating a control group of patients who did not experience the outcome of interest [22],[26]–[28].

### Identification of Case Patients

**Figure 1:**
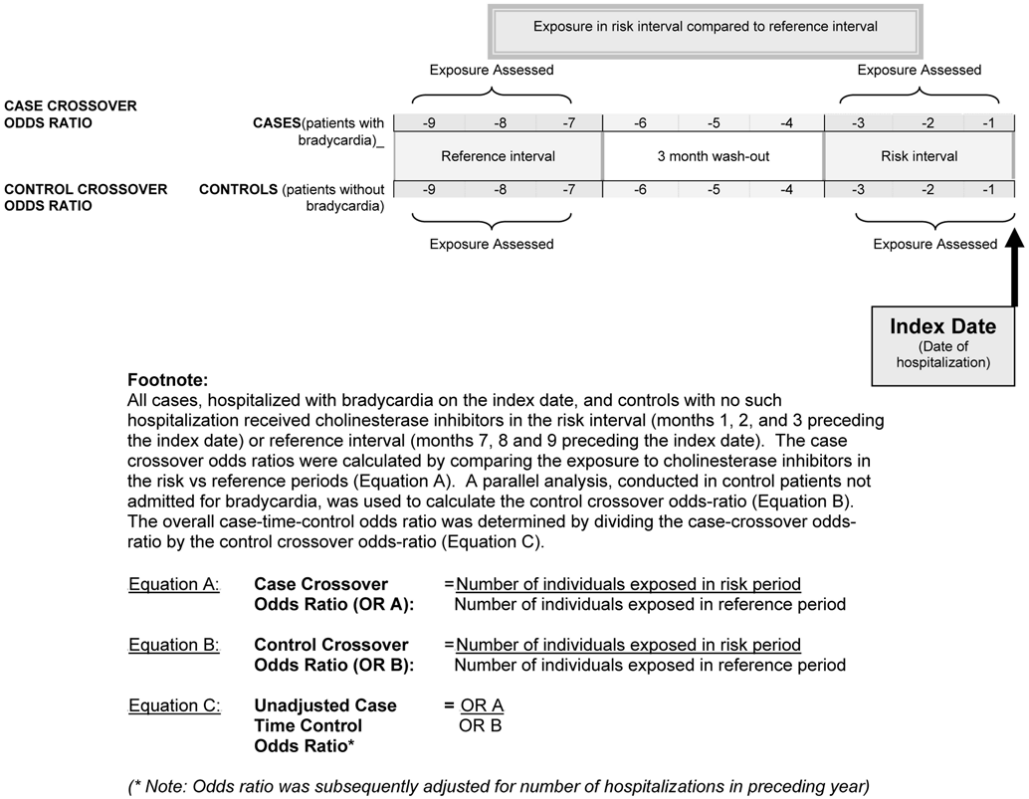
Case-time-control design.

We included all patients aged 67 y and older hospitalized with a diagnosis of bradycardia between January 1, 2003 and March 31, 2008, and restricted our analysis to those patients who were exposed to a cholinesterase inhibitor in the 9 mo prior to the index date. Because we hypothesized that bradycardia caused by cholinesterase inhibitors would be most likely to manifest during the initial period of therapy, we defined our risk interval as the 3-mo period immediately preceding hospitalization, and our reference interval as the months seven through nine prior to the index date (Figure 1). We included a 3-mo wash-out interval between the risk and reference intervals to avoid contamination between the risk and reference intervals, and excluded individuals with pacemaker insertion in the previous 5 y or hospitalization in the year preceding the study entry. Individuals with cholinesterase inhibitor prescription in the wash-out period, or both the risk and reference periods, did not contribute to the analysis.

Hospitalizations included emergency department visits and hospital admissions for bradycardia, and were identified using the International Classification of Diseases and Related Health Problems Tenth Revision (ICD-10) [29] code for bradycardia (R001). All hospital visits associated with a diagnosis of bradycardia were included in this study because the CIHI Discharge Abstract Database does not contain direct information on the primary reason for hospital admission. The date of the hospitalization served as the index date, and only the first hospitalization for bradycardia was considered for patients with multiple such admissions during the study period.

### Identification of Control Patients

In keeping with the case-time-control design, we corrected for temporal changes in cholinesterase inhibitor use by matching each case with up to three control patients. Control patients did not experience a hospitalization for bradycardia on or before the index date, but did receive at least one prescription for a cholinesterase inhibitor in either of the corresponding risk or reference intervals preceding the index date (Figure 1).

To minimize differences between case and control patients, we selected controls matched on age (born within 1 y of case), sex, and their anticipated risk of bradycardia using a disease risk index, as done previously [30],[31]. The disease risk index was derived by constructing a multivariable regression model that included multiple potential predictors of bradycardia or death, including socioeconomic status, residence in long-term care facility, overall number of prescription drugs prescribed in the preceding year, beta-blockers, calcium channel blockers, digoxin, antiarrhythmics, nitrates, anticoagulants, antiplatelets, diuretics, angiotensin-converting enzyme inhibitors, angiotensin receptor blockers, HMG-CoA reductase inhibitors, fibric acid derivatives, ezetimibe, oral hypoglycemic agents, insulin, antipsychotic medications, antidepressants, sedative-hypnotics, chemotherapy, corticosteroids, overall number physician clinic visits, emergency department visits, cardiologist visits, internist visits, neurologist visits, geriatrician visits, psychiatrist visits, coronary artery bypass graft, angiography, percutaneous transluminal coronary angioplasty, valve surgery, carotid endarterectomy, peripheral vascular disease procedures, dialysis, echocardiography, electrocardiography, holter monitor, nuclear medicine stress test, carotid doppler ultrasonography, charlson comorbidity index score, renal dysfunction, liver dysfunction, heart failure, diabetes, cancer, cerebrovascular disease (strokes, transient ischemic attacks), cardiac dysrhythmias, myocardial infarction, angina and coronary artery disease, peripheral vascular disease, major infections (respiratory, urogenital, abdominal, gastrointestinal, skin, soft tissue), and alcoholism (Text S1). These potential confounders were consequently summarized into a single disease risk index that predicted the probability of hospital admission for bradycardia [30],[32]. We selected up to three controls with the disease risk scores (within 0.2 standard deviation) closest to the given case.

### Statistical Analysis

We derived the case-time-control odds ratio [OR] by dividing the crossover OR among the cases (i.e., the case-crossover group) by the crossover OR among the controls (i.e., the control-crossover group), thereby producing a case-control OR adjusted for time-trend (Figure 1). Crossover ORs were derived from the ratio of discordant pairs, i.e., the number of individuals exposed exclusively during the risk interval as compared to exclusively during the reference interval [27]. By assuming conditional independence of exposure within each 1∶3 matched set, a conditional logistic regression model, adjusting for the length of stay in hospital in the year preceding study entry as an additional measure of comorbidity, was fitted to estimate the overall OR and 95% confidence intervals (CIs) [27].

We hypothesized that individuals with pre-existing cardiovascular disease and individuals co-using negative chronotropic agents might be at particularly high risk for bradycardia. To examine these risk groups, we performed a stratified analysis in those with a history of cardiovascular disease (defined as previous myocardial infarction, congestive heart failure, angina, or arrhythmias), and those coprescribed negative chronotropic medications such as beta-adrenergic antagonists, digoxin, or the nondihydropyridine calcium channel antagonists verapamil and diltiazem.

Drug interactions with cholinesterase inhibitors can occur via the cytochrome P450 enzyme 2D6. However, given the relatively few P450 2D6 inhibitors, we felt it was more relevant to focus on the pharmacodynamic interaction between cholinesterase inhibitors and negative chronotropic agents.

To test the specificity of our findings, we performed a sensitivity analysis in which proton pump inhibitors served as the exposure of interest rather than cholinesterase inhibitors. All analyses used a two-sided type I error rate of 0.05 and were performed using SAS version 9.1 (SAS Institute).

## Results

**Figure 2:**
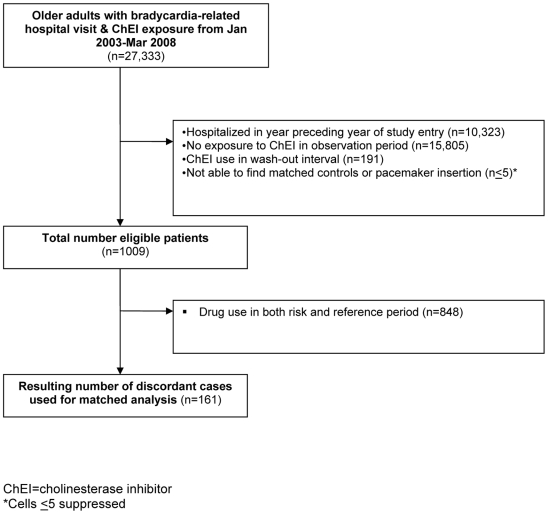
Flow diagram of case selection.

Between January 1, 2003 and March 31, 2008, we identified 27,333 hospitalizations for bradycardia among Ontario residents 67 y and older. Of these, 10,323 were excluded because they were hospitalized in the year prior to the index date, and 15,805 were excluded because they had not used cholinesterase inhibitors in the 9 mo prior to index date (Figure 2). Among the remaining patients, we further excluded 191 individuals exposed to cholinesterase inhibitors during the wash-out interval, and patients who had a pacemaker or could not be matched to at least one control (*n*≤5), leaving 1,009 eligible cases. Among these cases, 848 (84%) received a cholinesterase inhibitor in both the risk and reference periods, leaving 161 cases to inform our matched pairs analysis of individuals who had received a cholinesterase inhibitor in either the risk or reference period, but not both. Of these cases, 148 (92%) were fully matched to three controls and 157 (98%) were matched to at least two controls. We identified 466 matched controls from 42,833 potential controls.

The characteristics of cases and controls were highly similar (Table 1). The mean age of patients was 83 y (standard deviation 5.4 y) and 320 (51%) were female. A higher proportion of controls were in long-term care facilities, although the overall proportion remained low in both groups. Donepezil was the most frequently prescribed cholinesterase inhibitor in these patients accounting for 117 of the 161 (73%) cases and 292 of the 466 (63%) controls. Seventeen patients (11%) received a pacemaker during their hospitalization, and six (4%) individuals died prior to discharge from hospital.

**Table 1:** Characteristics of cases and controls.

Characteristic	Cases (*n* = 161)	Controls (*n* = 466)
**Age, mean (SD), y**	82.81±5.41	82.71±5.37
**Female**	82 (50.9%)	238 (51.1%)
**Low income**	42 (26.1%)	124 (26.6%)
**Charlson Index, mean score (SD)**	0.76±1.27	0.67±1.19
**Long-term care, preceding year**	8 (5.0%)	46 (9.9%)
**Drug therapy, preceding year**		
ACE inhibitor	58 (36.0%)	176 (37.8%)
Angiotensin receptor antagonist	18 (11.2%)	61 (13.1%)
Antiarrhythmic	≤5	18 (3.9%)
Anticoagulant	27 (16.8%)	70 (15.0%)
Antidepressant	33 (20.5%)	93 (20.0%)
Antiplatelet	28 (17.4%)	92 (19.7%)
Antipsychotic	9 (5.6%)	21 (4.5%)
Beta-adrenergic antagonist	53 (32.9%)	149 (32.0%)
Calcium channel antagonist	47 (29.2%)	145 (31.1%)
Corticosteroid	≤5	≤5
Digoxin	25 (15.5%)	63 (13.5%)
Diuretic	57 (35.4%)	156 (33.5%)
Fibric acid derivative	≤5	8 (1.7%)
Oral glucose lowering drug	21 (13.0%)	52 (11.2%)
Insulin	≤5	12 (2.6%)
Nitrate	18 (11.2%)	55 (11.8%)
Sedative hypnotic	22 (13.7%)	72 (15.5%)
Statin	41 (25.5%)	123 (26.4%)
**Total ** ***n*** **drugs prescribed in preceding year, median (IQR)**	8 (5–12)	8 (5–12)
**Total** ***n*** ** physician visits in preceding year**		
**Cardiologist**		
Mean±SD	0.52±1.06	0.44±1.51
Median (IQR)	0 (0–1)	0 (0–0)
**Family physician**		
Mean±SD	13.88±13.88	13.75±12.07
Median (IQR)	10 (5–18)	11 (6–18)
**Geriatrician**		
Mean±SD	0.17±0.84	0.17±0.68
Median (IQR)	0 (0–0)	0 (0–0)
**Internist**		
Mean±SD	2.20±4.71	1.96±4.90
Median (IQR)	1 (0–2)	1 (0–2)
**Neurologist**		
Mean±SD	0.08±0.33	0.08±0.36
Median (IQR)	0 (0–0)	0 (0–0)
**Psychiatrist**		
Mean±SD	0.36±3.19	0.45±2.90
Median (IQR)	0 (0–0)	0 (0–0)
**Total** ***n*** ** emergency department visits, preceding year**	0.73±1.65	0.77±1.71
**Total ** ***n*** ** medical visits, preceding year**	22.81±19.62	22.19±17.81
**Total ** ***n*** ** days stayed in hospital, preceding year**	2.02±6.27	1.95±7.29
**Medical conditions, preceding 5 y**		
Heart failure	28 (17.4%)	90 (19.3%)
Myocardial infarction	74 (46.0%)	230 (49.4%)
Peripheral vascular disease	≤5	≤5
Alcoholism	≤5	19 (4.1%)
Angina	49 (30.4%)	160 (34.3%)
Arrhythmia	15 (9.3%)	31 (6.7%)
Diabetes	42 (26.1%)	109 (23.4%)
Liver disease	≤5	11 (2.4%)
Renal dysfunction	57 (35.4%)	146 (31.3%)
Stroke	41 (25.5%)	112 (24.0%)
**Medical procedures, preceding 5 y**		
Coronary artery bypass graft	≤5	7 (1.5%)
Percutaneous transluminal coronary angioplasty	7 (4.3%)	19 (4.1%)
Peripheral vascular disease	0 (0.0%)	≤5
Angiography/cardiac catheterization	9 (5.6%)	23 (4.9%)
Carotid doppler ultrasound	35 (21.7%)	107 (23.0%)
Carotid endarterectomy	≤5	≤5
Dialysis	0 (0.0%)	0 (0.0%)
Echocardiogram	70 (43.5%)	212 (45.5%)
Electrocardiography	144 (89.4%)	418 (89.7%)
Holter monitoring	52 (32.3%)	141 (30.3%)
Stress and nuclear tests	50 (31.1%)	147 (31.5%)
Valve surgery	0 (0.0%)	0 (0.0%)

All data presented as number (percentages) except where indicated. Cells ≤5 are suppressed.

ACE, angiotensin-converting enzyme; IQR, interquartile range; SD, standard deviation.

In the primary analysis, recent initiation of cholinesterase inhibitors was significantly associated with hospitalization for bradycardia (adjusted OR 2.13, 95% CI 1.29–3.51, *p* = 0.003; Table 2). Among cases and controls with previously diagnosed cardiac disease, the association between recent use of cholinesterase inhibitors and bradycardia was similar (adjusted OR 2.25, 95% CI 1.18–4.28, *p* = 0.014). The association persisted among the cases and controls receiving negative chronotropic medications (adjusted OR 2.34; 95% CI 1.16–4.71, *p* = 0.017). As expected, we found no association between recent initiation of proton pump inhibitors and bradycardia (adjusted OR 1.13, 95% CI 0.93–1.37, *p* = 0.228).

**Table 2:** Risk of bradycardia-related hospital admissions and recent cholinesterase inhibitor use.

Overall and Subgroup Analyses	Exposure in Risk Interval	Exposure in Reference Interval	Adjusted OR^a^ (95% CI)
**Full population**			
Overall			2.13 (1.29–3.51)
Cases (*n* = 161)	139	22	*p* = 0.003
Control (*n* = 466)	349	117	
**I. Subgroup with cardiac comorbidity**			
Overall			2.25 (1.18–4.28)
Cases (*n* = 97)	84	13	*p* = 0.014
Control (*n* = 274)	202	72	
**II. Subgroup using negative chronotropes**			
Overall			2.34 (1.16–4.71)
Cases (*n* = 80)	69	11	*p* = 0.017
Control (*n* = 220)	158	62	

a Case-time-control OR adjusted for hospitalization length of stay in the preceding year.

### Secondary Analyses

Because bradycardia is a relatively common occurrence in older patients, we hypothesized that the potential contribution of cholinesterase inhibitors to the development of bradycardia might not be recognized, and that therapy might be continued following discharge. After excluding individuals who received pacemakers during their hospital stay, 138 cases survived to discharge. Of these cases, subsequent prescription records indicated that cholinesterase inhibitors were restarted in 78 individuals (57%) within 100 d of hospital discharge. A post hoc examination of the 3-mo period following resumption of therapy in those 78 individuals showed that three (3.8%) individuals were readmitted to hospital or visited the emergency department with a diagnosis of bradycardia.

## Discussion

Using the health care records of more than 1.4 million Ontario residents aged 67 y and older, we found that treatment with cholinesterase inhibitors was associated with a doubling in the risk of hospitalization for bradycardia. Importantly, although cholinesterase inhibitors are reversible precipitants of bradycardia, the drugs were resumed following discharge in greater than half the cases, presumably because the potential causative role of these drugs in the hospitalization was not appreciated. In many patients, cholinesterase inhibitors are associated with marginal improvement in cognition and global functioning [1],[33]. Consequently, recent guidelines suggest that cholinesterase inhibitors should not be standard of care for patients with dementia, but instead urge physicians to weigh each individual's expected risks and benefits before initiating therapy. Our large-scale population-based assessment of the risk of bradycardia with cholinesterase inhibitors in clinical practice should help inform the risk-benefit assessment for clinicians and patients. A recent study used an alternate cohort design to examine a primary outcome of syncope in patients receiving cholinesterase inhibitors. In a secondary analysis, these investigators also observed an elevated risk of bradycardia (adjusted hazard ratio 1.69; 95% CI 1.32–2.15) associated with cholinesterase inhibitor use, which complements the findings of our study [34].

The risk of bradycardia observed in our study may cause clinicians and patients to reconsider therapy with these drugs, particularly for patients in whom little or no cognitive improvement is observed early in therapy [35],[36]. At a minimum, our findings should alert clinicians to the potential role of cholinesterase inhibitors in patients with bradycardia, for whom resumption of treatment may be inadvisable.

Our study has several limitations that merit emphasis. Administrative databases contain information on hospitalizations, emergency department visits, diagnoses, procedures, physicians' claims, outpatient clinic visits, and drug dispensing data. However, they lack the clinical richness of a medical chart. Accordingly, we were not able to capture the severity of dementia, lifestyle habits such as smoking, diet, alcohol use, exercise, over-the-counter drug use, in-hospital drug use, laboratory values, and the results of diagnostic testing.

However, we relied on the unique crossover design feature that controls for fixed patient characteristics by allowing each patient to serve as his or her own control. Consequently, concerns regarding potential confounders, including disease severity, were largely overcome. The crossover design also relies on the temporal association between an exposure and outcome, and this feature enabled us to examine whether bradycardia-related hospitalization was likely secondary to cholinesterase inhibitors as opposed to underlying comorbidities. More specifically, the crossover design examines whether an exposure is more likely to occur immediately preceding an event or during another period when bradycardia did not occur. If the exposure is not associated with the outcome, then no temporal association would be expected. The presence of underlying comorbidities would not be expected to confound the cholinesterase inhibitor–bradycardia relationship unless the comorbidities were associated with both cholinesterase inhibitor use and bradycardia. To further control for confounding, we corrected for temporal changes in cholinesterase inhibitor use by matching each case with up to three control patients on the basis of an extensive disease risk index score.

We identified episodes of bradycardia resulting in emergency department visits or hospital admissions, but we were unable to identify individuals in whom bradycardia did not culminate in hospital care, including cases in which bradycardia led to death [37]. Therefore, our analysis likely underestimates the true risk of cardiovascular harm associated with cholinesterase inhibitors. The coding for bradycardia has not been validated and it is possible that some cases of bradycardia were missed if the code for bradycardia has low sensitivity. However, we expect that the positive predictive value for the bradycardia code would be reasonable because the diagnosis for bradycardia is based upon a fairly straightforward medical assessment. The occurrence of random miscoding would only have attenuated our estimates and biased the results towards the null. It is likely that the cases of bradycardia captured in our study were clinically significant because they were severe enough to be documented in the patient's chart and coded in the CIHI database.

We were unable to assess the absolute risk of bradycardia due to cholinesterase inhibitor therapy, and we could not be certain that resumption of cholinesterase inhibitors following hospital discharge truly reflected a lack of appreciation for the potential negative chronotropic effects of therapy. Since most patients were taking donepezil, we were not able to contrast risks with individual cholinesterase inhibitors given the low prevalence of use with the other agents. Future studies are needed to address the relative harms of the individual drugs in this class.

Our post hoc examination of the 78 patients who resumed cholinesterase inhibitor after hospital discharge showed that only 4% were readmitted to hospital or visited the emergency department with a diagnosis of bradycardia in the 3 mo following resumption of therapy. It is hard to know how to interpret this post hoc analysis. We did not evaluate out-of-hospital death, and could not ascertain whether cholinesterase inhibitor therapy was restarted with a reduced dose or more gradual dose titration, whether changes to other medications were made, or whether closer outpatient follow up for bradycardia was undertaken.

Finally, because we relied on drug dispensing data as a proxy for drug adherence, there was a potential for exposure misclassification.

While bias and confounding can threaten any observational study, the likelihood of these was reduced substantially by the design of our study, in which every case served as his or her own control, thereby lessening between-patient variability, and by the use of a disease risk index, an advanced matching technique, which resulted in considerable similarity between cases and controls (Table 1) [31],[32].

In summary, we found that cholinesterase inhibitors are associated with a significantly increased risk of hospitalization for bradycardia among older outpatients, and that the risk is similar in patients with cardiovascular comorbidity and those receiving concurrent therapy with negative chronotropic drugs. Our findings highlight the importance of careful clinical evaluation prior to initiating cholinesterase inhibitor therapy, and vigilant monitoring thereafter in order to prevent adverse cardiac events. Cholinesterase inhibitors should be prescribed judiciously, and because these drugs carry a risk of serious adverse events, they should be continued only if the benefits outweigh the risks. Finally, it is important for clinicians to be aware of the potential negative chronotropic effects of cholinesterase inhibitors, and reassess the merits of continued therapy in patients who develop bradycardia while taking these drugs. The frequent resumption of cholinesterase inhibitors following discharge suggests that bradycardia may not be widely recognized as a potential adverse effect of this class of medications.

## Supporting Information

Text S1 Variables used in the Disease Risk Index.
(0.06 MB DOC)

## Abbreviations

CI: confidence interval
CIHI: Canadian Institute for Health Information
OR: odds ratio
