# Supplementary material for: Cholinesterase Inhibitors and Hospitalization for Bradycardia: A Population-Based Study
Source: PLoS Med. 2009 Sep 29;6(9):e1000157. doi: 10.1371/journal.pmed.1000157 (PMC2742897; doi:10.1371/journal.pmed.1000157)
Supplement: Text S1 — Variables used in the Disease Risk Index. (0.06 MB DOC) [file pmed.1000157.s001.doc]

**Appendix 1: Variables used in the Disease Risk Index**

| Demographic | Age |
| --- | --- |
|  | Sex |
|  | Socioeconomic status |
|  | Residence in long-term care facility |
|  |  |
| Treatments | (Dispensed within preceding 90 days) * |
|  | Number of prescription drugs prescribed in last year |
|  | Beta-blockers |
|  | Calcium channel blockers |
|  | Digoxin |
|  | Antiarrhythmics |
|  | Nitrates |
|  | Anticoagulants |
|  | Antiplatelets |
|  | Diuretics |
|  | Angiotensive converting enzyme inhibitors |
|  | Angiotensin receptor blockers |
|  | HMG-CoA reductase inhibitors (statins) |
|  | Fibric acid derivatives |
|  | Ezetimibe |
|  | Oral hypoglycemic agents |
|  | Insulin |
|  | Antipsychotic medications |
|  | Antidepressants |
|  | Sedative hypnotics |
|  | Chemotherapy |
|  | Corticosteroids |
|  |  |
| Resource Utilization | (Within preceding 1 year) * |
|  | Ontario Health Insurance Plan clinic visits (any medical visit) |
|  | Emergency department visits |
|  | Cardiologist visits |
|  | Internist visits |
|  | Neurologist visits |
|  | Geriatrician visits |
|  | Psychiatrist visits |
|  |  |
| Procedures | (Within preceding 5 years) * |
|  | Coronary artery bypass graft |
|  | Angiography |
|  | Percutanueous transluminal coronary angioplasty |
|  | Valve surgery |
|  | Permanent pacemaker insertion |
|  | Carotid endarterectomy |
|  | Peripheral vascular disease procedures |
|  | Dialysis |
|  | Echocardiography |
|  | Electrocardiography |
|  | Holter monitor |
|  | Nuclear medicine stress test |
|  | Carotid Doppler ultrasonography |
|  |  |
| Comorbidities | (Within preceding 5 years) * |
|  | Charlson comorbidity index score |
|  | Renal dysfunction |
|  | Liver dysfunction |
|  | Heart failure |
|  | Diabetes |
|  | Cancer |
|  | Cerebrovascular disease (strokes, transient ischemic attacks) |
|  | Cardiac dysrhythmias |
|  | Myocardial infarction |
|  | Angina and coronary artery disease |
|  | Peripheral vascular disease |
|  | Major infections (respiratory, urogenital, abdominal, gastrointestinal, skin, soft tissue) |
|  | Alcoholism |

***** Prior to 1 year pre-index
